# Tumor immunology and cancer immunotherapy: summary of the 2013 SITC primer

**DOI:** 10.1186/2051-1426-2-14

**Published:** 2014-05-14

**Authors:** Raju R Raval, Andrew B Sharabi, Amanda J Walker, Charles G Drake, Padmanee Sharma

**Affiliations:** 1 Department of Radiation Oncology & Molecular Radiation Sciences, Johns Hopkins University School of Medicine, Baltimore, MD, USA; 2 Department of Medical Oncology, Johns Hopkins University School of Medicine, Baltimore, MD, USA; 3 The Brady Urological Institute, Johns Hopkins University School of Medicine, Baltimore, MD, USA; 4 Department of Genitourinary Medical Oncology, The University of Texas MD Anderson Cancer Center, Houston, TX, USA; 5 Department of Immunology, The University of Texas MD Anderson Cancer Center, Houston, TX, USA

**Keywords:** CTLA-4, PD-1, Melanoma, Prostate cancer, Kidney cancer, Bladder cancer, PD-L1, LAG-3

## Abstract

Knowledge of the basic mechanisms of the immune system as it relates to cancer has been increasing rapidly. These developments have accelerated the translation of these advancements into medical breakthroughs for many cancer patients. The immune system is designed to discriminate between self and non-self, and through genetic recombination there is virtually no limit to the number of antigens it can recognize. Thus, mutational events, translocations, and other genetic abnormalities within cancer cells may be distinguished as “altered-self” and these differences may play an important role in preventing the development or progression of cancer. However, tumors may utilize a variety of mechanisms to evade the immune system as well. Cancer biologists are aiming to both better understand the relationship between tumors and the normal immune system, and to look for ways to alter the playing field for cancer immunotherapy. Summarized in this review are discussions from the 2013 SITC Primer, which focused on reviewing current knowledge and future directions of research related to tumor immunology and cancer immunotherapy, including sessions on innate immunity, adaptive immunity, therapeutic approaches (dendritic cells, adoptive T cell therapy, anti-tumor antibodies, cancer vaccines, and immune checkpoint blockade), challenges to driving an anti-tumor immune response, monitoring immune responses, and the future of immunotherapy clinical trial design.

## Introduction

The innate and adaptive immune systems function to protect the host from foreign pathogens, and are generally tolerant toward host tissues – adequately differentiating between “self” and “non-self” antigens. In the setting of an evolving tumor, the immune system is likely exposed to numerous, previously unseen, antigens arising from genetic abnormalities. Interestingly, it is thought that the immune system is able to perceive and eliminate some tumors early on in their development. However, the theory of immunoediting, which involves the process of immunosurveillance, suggests that certain tumors escape from an equilibrium state previously held in check by the immune system, and become clinically significant [1]. Oncologists and cancer researchers are focused on understanding these mechanisms, and in finding novel (often combinatorial) approaches to cancer immunotherapy. There are a variety of approaches to eliciting an anti-tumor immune response, with advancements in techniques involving therapeutic cancer vaccines, adoptive T cell therapy, anti-tumor antibodies, and immune checkpoint blockade. In addition, combining these approaches with other therapies such as immunomodulators (cytokines, cyclic dinucleotides, IDO inhibitors), cytotoxic chemotherapy, radiation therapy, or molecularly targeted therapies may hold the key to the true potential of immunotherapy in the future management of cancer patients.

In its desire to further explore and educate the broader scientific community on these advancements, the Society for Immunotherapy of Cancer (SITC) convened a Primer on Tumor Immunology and Cancer Immunotherapy, which was organized by Padmanee Sharma, MD, PhD and Charles Drake, MD, PhD. Experts in the fields of tumor immunology led lectures and discussions on a variety of topics including Innate Immunity (Vincenzo Bronte, MD), Dendritic Cells (A. Karolina Paluka, MD, PhD), Adoptive Immunity (Jonathan Powell, MD, PhD), Adoptive T cell Therapy (Cassian Yee, MD), Anti-tumor Antibodies (Charles Drake, MD, PhD), Challenges to Driving an Immune Response (Jedd Wolchok, MD, PhD), Cancer Vaccines (Nina Bhardwaj, MD, PhD), Immune Checkpoint Blockade (James Allison, PhD), Monitoring Immune Responses (Sacha Gnjatic, PhD), and Immunotherapy Clinical Trial Design (Padmanee Sharma, MD, PhD).

## Review

### Innate immunity

The innate immune system acts as a first line of defense against foreign pathogens, responds over a short period of time within minutes to hours, has a variety of effector mechanisms, and is both phylogenetically older than and can shape the adaptive immune response. There are a multitude of diverse components of innate immunity including physical barriers (skin epithelium and mucosal membranes), effector cells (macrophages, NK cells, innate lymphoid cells, dendritic cells, mast cells, neutrophils, and eosinophils among others), mechanisms of pattern recognition (Toll-like receptors), and humoral mechanisms (complement proteins or cytokines). In contrast to the more specific, but slower adaptive immune response consisting primarily of B and T cells, the more rapid innate immune response is usually characterized by tissue inflammation (with physical characteristics manifested usually by heat, pain, swelling, and erythema). Tissue inflammation as part of the innate immune response serves to help eliminate invasive foreign pathogens, initiate tissue repair, and can serve to stimulate the adaptive immune response through B and T cells. However, there is a significant amount of evidence that both acute and chronic inflammation may promote genetic abnormalities and cancer progression.

In an environment of chronic inflammation, myeloid cell differentiation can be skewed toward the expansion of myeloid-derived suppressor cells (MDSCs). MDSCs are a heterogeneous population of myeloid derived immune cells (including macrophages, neutrophils, and dendritic cells) that can have potent immunosuppressive activities [2]. Among these effects, MDSCs can inhibit T cell proliferation and activation. In regions of inflammation such as tumors, these cells can inhibit anti-tumor immune responses through suppression of both T cells and NK cells [2]. More broadly it has been shown that MDSCs can promote neoangiogenesis, tumor stromal remodeling, and even metastasis. Interestingly, mutations within tumors, such as BRAF mutations, can drive these cells to produce pro-inflammatory cytokines such as IL-6, IL-10, and VEGF that have been implicated in MDSC development and regulation [2,3]. Furthermore, signals mediated through members of the signal transducer and activator of transcription (STAT1, STAT3, STAT6) and NF-kB (nuclear factor kappa-light-chain enhancer of activated B cells) transcription factors can stimulate MDSCs to suppress anti-tumor immune responses [2,4]. Thus, finding ways to either decrease chronic inflammation or more specifically exploring paths to limit the function of MDSCs are intense areas of research. Indeed, a number of currently used clinical pharmacologic agents (PDE5 inhibitors, COX-2 inhibitors, ARG1 inhibitors, bisphosphonates, gemcitabine, and paclitaxel among others) along with other agents in preclinical testing may play a profound role in promoting anti-tumor immune responses by inhibiting the function or proliferation of MDSCs [5]. Thus, further understanding and finding ways to modulate the innate immune system may play a major role in the future of cancer immunotherapy.

### Dendritic cells

Professional antigen presenting cells include Dendritic Cells (DCs), Macrophages, and B-cells. Of these DCs are the most potent antigen presenters given their morphologic and phenotypic properties. DCs in the skin were initially discovered by Paul Langerhans and were termed dendritic cells by Ralph Steinman due to the numerous dendrites which serve to increase the surface area for antigen presentation and cell-cell interactions [6]. Important for their function, these dendrites facilitate high concentrations of MHC-antigen complexes and cell surface co-stimulatory molecules required for robust T-cell activation. In this way, DCs serve as a key link between the innate and adaptive arms of the immune system. DCs can develop from either myeloid or lymphoid hematopoietic lineages, which can thus give rise to different subsets of DCs with varying functions. Furthermore, DCs can have different effector functions depending on their tissue of residence and microenvironment. Langerhans cells are a subset of DCs which reside in the epidermal layers of the skin and function to continuously patrol and scan for pathogens [6]. Langerin negative dermal DCs are a subset residing in the dermis and also play a key role in generating cellular immunity. Interstitial CD14+ DCs are thought to be less efficient at activating naïve T-cells and have proven tolerogenic functions [7]. Plasmacytoid DCs are the most frequent DCs in the blood and play a key role in secretion of Type I Interferons upon encounter with viruses [8]. Importantly, a subset of CD8+ DCs in mice and CD141+ DCs in humans have been reported to play a major role in cross-presentation and priming of anti-tumor immune responses [9,10]. Of note, bone marrow derived DCs (BMDC) or peripheral blood derived DCs (PBDC) generated by culture of monocytes in IL-4 and GM-CSF are commonly used for research, and as vaccines in clinical trials. Recently, DC cell lines have been developed which may facilitate further research into the mechanisms of DC function [11]. However, there remain important phenotypic differences between mouse and human DC subsets which should not be overlooked [12].

In the absence of pathogens, DCs are generally in a resting state. However, upon encountering inflammatory 'danger' signals, pathogen associated molecular patterns (PAMPs), phagocytosis of pathogens, or pinocytosis of pro-inflammatory cellular debris, DCs undergo an orchestrated activation and maturation process which is critical for their function. The toll-like receptor (TLR) family recognizes a multitude of different PAMPs, and the specific TLR which is activated can skew towards an effective immune response to counter that specific pathogen [13]. Activation of DCs increases their cell-surface expression of MHC class I, MHC class II, CD80, CD86, and additional positive and negative co-stimulatory surface molecules. Additionally, DCs can secrete a myriad of cytokines and chemokines (including interferons, TNF alpha, IL-1, IL-4, IL-6, IL-10, IL-12, and IL-23) that can guide and skew resultant T-cell responses [14]. For example, signaling through TLR4 generally results in production of IL-12, which can skew towards a T_H_1 T cell phenotype while signaling through TLR6 can result in IL-10 production skewing towards a T_H_2 T cell phenotype [15,16]. Upon activation, certain DCs up-regulate specific cell-adhesion molecules which facilitate migration from their tissue of residence back to a lymph node or lymphoid follicle to present antigen to residing T-cells. Importantly, the type of maturation process that the DC goes through can have a distinct effect on the immune response that is elicited.

Given that DCs are such potent antigen presenting cells, there has been significant effort aimed at inducing anti-tumor immune responses using DCs. DC based immunotherapy aims to induce an effective anti-tumor response using externally generated DCs as antigen presenting cells. The widespread implementation of this approach has been hampered by logistical challenges and limited clinical success. However, the first FDA approved DC-based immunotherapy (sipuleucel-T) was approved in 2010 for men with metastatic castrate resistant prostate cancer. This cell-based vaccine uses PBMCs incubated with a Prostatic Acid Phosphatase GM-CSF fusion protein that drives the maturation of monocytes to dendritic cells. In a randomized controlled trial in men with castrate resistant prostate cancer, treatment with sipuleucel-T resulted in a 4.1 month improvement in median survival compared to placebo (HR 0.78; p = 0.03) [17]. Overall, this targeted immunotherapy has a favorable safety profile with the main side effects limited to chills, fever, and headache.

Recent efforts have focused on enhancing the potency of DCs with the goal of increasing the magnitude and duration of an induced anti-tumor immune responses using 2nd and 3rd generation DC vaccines. One strategy is to target antigens to specific subsets of DCs primed to induce the immune response of interest. Another strategy is to use specific DC agonists such as anti-CD40 to drive DC generated immune responses in-vivo [18,19]. Finally, antigen presentation attenuators, such as SOCS1 and A20 have been shown to restrict the ability of DCs to induce a potent immune response in part by blocking secretion of critical cytokines. A novel strategy of inhibiting these negative regulators using siRNA technology may be key to removing the brakes on DCs and unlocking their full potential for immunotherapy [20,21].

### The adaptive immune system

The adaptive arm of the immune system consists of B cells and T cells. In contrast to the innate immune response which recognizes pathogens based on nonspecific molecular patterns, such as single stranded RNA or lipopolysaccharide, the adaptive immune response is driven by a vast array of incredibly diverse and highly specific antigen receptors on T cells (TCR) and B cells (BCR). The diversity and specificity of these antigen specific receptors are a result of V(D)J recombination, a form of genetic recombination that randomly combines Variable, Diversity, and Joining gene segments, and allows the generation of millions of different highly specific receptors. An effective immune response is initiated when a B cell or T cell recognizes antigen in a pro-stimulatory context, and undergoes selective activation and proliferation. This proliferative process, as a result of activation, is known as clonal selection and promotes robust antigen-specific immune responses as well as the development of long-lasting memory cells. The latter phenomenon allows a more rapid and robust immune response upon re-exposure to the same pathogen. Understanding how the adaptive arm of the immune system is engaged and regulated requires knowledge of both B cell and T cell biology.

The binding moiety of the B cell receptor (BCR) is a cell-surface immunoglobulin that is able to recognize soluble antigen based on its unique antigen-binding site. Once the BCR is cross-linked by specific, soluble antigen, the cell undergoes growth, division, and further differentiation into a plasma cell. This leads to the proliferation of a pool of plasma cells from the same clone that collectively secrete large amounts of highly specific antibodies, in a process similar to the clonal selection of T cells. Briefly, antibodies are Y-shaped glycoproteins containing a variable (antigen binding) and constant region. The constant region specifies the class of antibody (in humans: IgA, IgD, IgE, IgG, and IgM). Antibodies have several effector mechanisms including neutralization of antigen, agglutination of microbes, precipitation of dissolved antigens, activation of the complement cascade, and antibody-dependent cellular cytotoxicity (ADCC).

Unlike B cells and gamma-delta T cells, which can detect soluble antigen, classical T cells (alpha-beta) recognize antigen in the form of small peptides presented by antigen-presenting cells in the context of MHC molecules on the cell surface. Intracellular antigens are proteolyzed and representative peptides are “presented” on the surface of cells complexed within the groove of the MHC molecule. T cells then detect antigen bound to MHC molecules on cell surfaces. There are two major classes of T cells and MHC molecules. The CD4+ T cell subset binds to antigen presented by MHC class II molecules, which are primarily expressed by antigen presenting cells (APCs). CD8+ T cells bind to MHC class I molecules, which are present on every nucleated cell, including APCs. After a naïve CD4+ T cell is engaged by an APC, it further differentiates into one of many types of CD4+ effector cells depending on the cytokine milieu of the microenvironment during activation. One path is to differentiate into a T helper cell and release cytokines that “help” activate B cells, NK cells, and CD8+ cytotoxic lymphocytes. Different types of T helper cells exist that have distinct roles depending on the pathogen and type of immune response being generated (T_H_1, T_H_2, T_H_17, etc.). When a CD4+ T cell recognizes antigen in the context of a non-inflamed environment (TGF-beta, IL-10), it can differentiate into a regulatory T cell (Treg). Tregs are important negative regulators of the immune system and are a robust target for cancer immunotherapy because they are often present within tumor tissue and are known to inhibit the host’s adaptive and innate anti-tumor immune response. In contrast to CD4+ “helper” T cells, CD8+ T cells are responsible for direct cell mediated cytotoxicity following activation by APCs and are thought to play a central role in the anti-tumor immune response. After being activated and exerting their cytotoxic effects, most CD8+ T cells undergo programmed cell death as a compensatory mechanism to prevent over-activation of the immune system and thus limiting potential collateral damage. However, a minority (5-10%) of the activated terminally differentiated CD8+ T cells will become long-lived memory cells. These T cells demonstrate enhanced functional responses upon re-exposure to antigen as compared to naïve T cells. Eliciting a memory response is an aspirational goal of cancer immunotherapy because the presence of memory cells potentially limits tumor regrowth and metastatic spread, even months to years after eradication of clinically evident disease.

The adaptive immune response is tightly regulated by multiple costimulatory and coinhibitory pathways. As previously described, binding of the TCR to the antigen/MHC complex (signal 1) is required for the activation of naive T cells. However, signal 1 is not sufficient to generate and maintain an adaptive immune response. Full activation of a T cell also requires the simultaneous engagement of positive costimulatory molecules present on activated APCs, known as signal 2. These costimulatory molecules are not present on quiescent APCs, tumor cells, or normal host cells. A classic example of a costimulatory pathway is the interaction between B7 expressed on an APC and CD28 expressed on T cells [22]. When the MHC/antigen complex and the TCR (signal 1), and the costimulatory pathway (signal 2) are activated, there is proliferation and activation of T cells. Since both costimulatory and coinhibitory molecules may be present at the same time, signal 2 is perhaps more appropriately conceptualized as the sum of both costimulatory signals and coinhibitory signals that determine T cell phenotype. After activation, T cells express coinhibitory receptors such as CTLA-4, PD-1, and LAG-3. These compensatory coinhibitors attenuate the immune response and are often co-opted by tumors to evade the host’s natural anti-tumor immune response.

The generation of antigen receptor diversity is a stochastic post-germline event, involving somatic recombination of gene segments, and is necessary to deal with the vast array of potential pathogens. However, there must be mechanisms in place to prevent these receptors from identifying and reacting with host tissues. Collectively, immunologic self tolerance is generated by the processes of central and peripheral tolerance. In broad terms, central tolerance involves the process of clonal deletion of auto-reactive T cells in the thymus, while peripheral tolerance incorporates multiple mechanisms of suppressing immune responses in tissues outside the thymus and bone marrow. For example, one form of tolerance is known as anergy, and occurs when a T cell receptor recognizes its cognate antigen in the absence of appropriate costimulatory molecules (i.e. only signal 1 is transmitted). This is often the case for CD8 T cell recognition of tumor cells, because tumor cells do not express the appropriate costimulatory molecules on their surface. Alternatively, activated T cells that receive repeated excessive stimulation via signal 1 with or without signal 2 may develop an “exhausted” phenotype becoming incapable of further activation or division even when exposed to antigen in pro-stimulatory conditions. In cancer immunity, APCs may also play a role in tolerance by presenting antigens in a tolerogenic environment leading to a lack of T cell activation and potential exhaustion. Exhausted T cells are usually incapable of activation, even in the presence of a fully activated APC. Similar to T cell differentiation and maturation, T cell activation is also clearly influenced by the microenvironment during antigen recognition, and the net sum of signals dictates the immune response. Potential strategies for inducing sustained anti-tumor immune responses often focus on modifying both costimulatory and inhibitory pathways. The ideal cancer immunotherapy would elicit a highly specific and durable immune response – two features unique to adaptive immunity. Further understanding of immune tolerance and the array of costimulatory and inhibitory signaling networks that regulate anti-tumor immune responses will be critical in order to optimize current treatment strategies and to promote the discovery of novel immunomodulating agents.

### Adoptive T cell therapy

Adoptive T cell therapy (ACT) is a promising and rapidly advancing form of immunotherapy that overcomes tolerance by harnessing the natural ability of immune cells to recognize and eliminate target cells in order to generate durable anti-tumor immune responses. Adoptive T cell therapy involves the infusion of externally manipulated T cells. There are multiple sources and types of T cells used for adoptive therapy, including expanded and activated tumor infiltrating lymphocytes (TILs), T cells modified ex-vivo to express a specific TCR, and T cells engineered to express a receptor that is a fusion between an antibody and the T cell receptor’s intracellular machinery, a so-called chimeric antigen receptor, or CAR [23].

The potential to treat metastatic solid tumors via manipulation of endogenous T cells was first explored in the early 1990s with the use of high dose intravenous interleukin-2 (IL-2), a canonical T cell growth factor [24]. High dose IL-2 was FDA approved in 1992 and leads to complete durable responses in 5-10% of patients with metastatic melanoma and renal cell cancer [25,26]. Building on this success, innovative treatment strategies using tumor-infiltrating lymphocytes (TILs) were developed. TIL therapy involves extracting lymphocytes from tumor tissue, *ex vivo* expansion with IL-2 followed by reinfusion [27]. A recent pooled analysis of TIL protocols reported a 20% complete response rate and a 70% overall objective response rate in patients with melanoma [28]. Prior to T cell infusion, patients receive non-myeloablative leukoreductive therapy (e.g. cyclophosphamide and fludarabine, with or without total body irradiation) in order to promote homeostatic proliferation of the infused T cells. After infusion, patients require maintenance therapy with high dose IL-2. Serious adverse events were seen in these trials including uveitis, PCP pneumonia, and respiratory compromise requiring intubation. Although expanded TILs are thought to be one of the least labor-intensive ACT strategies, several limitations preclude widespread adoption at the current time. These include the need for appropriate cell processing equipped facilities as well as the need for patients to have moderately bulky tumors for TIL isolation. Another approach to adoptive T cell therapy is the use of endogenous peripheral tumor specific T cells that are specifically expanded and activated *ex vivo* with reintroduction into the host via adoptive transfer [29-31]. This approach is somewhat labor intensive, involving multiple pheresis sessions to isolate PBMCs followed by the expansion of antigen-specific T cells.

Multiple approaches have been explored in an effort to expand the use of ACT to cancer types other than melanoma. One of the most promising strategies is to administer T cells that have been genetically engineered to express tumor-specific antigen receptors. These may be traditional TCRs that recognize epitopes of intracellular antigenspresented by MHC molecules, or chimeric antigen receptors (CARs) that include an extracellular antibody single-chain variable region joined with the intracellular portion of a TCR. CARs are unique in that they combine the cytotoxic activity of a CD8+ T cell with the highly avid and MHC-independent antigen recognition capacity of high-affinity monoclonal antibodies. To help overcome tolerance mechanisms, second generation CARs include expression of co-stimulatory signaling domains in addition to the CAR. There have been promising clinical results with refractory chronic lymphocytic leukemia (CLL) using a lentiviral derived vector expressing a CAR with specificity for CD19 (a B cell antigen) [32]. This CAR is coupled with two signaling domains including the cytoplasmic domain of 4-1BB receptor (CD137), which serves as a costimulatory receptor in T cells, and CD3-zeta, a signal-transduction component of the T cell antigen receptor. Two of three patients with CLL treated with this regimen demonstrated a complete remission, and a portion of the transformed T cells expressing the CAR persisted as memory T cells that retained CD19 effector functionality [32]. Unlike TIL therapy which often leads to widespread systemic toxicity, the grade 3 or 4 toxicities experienced in this clinical series were tumor lysis syndrome with associated cytokine release and lymphopenia. However, not unexpectedly, patients experience chronic B cell aplasia and hypogammaglobulinemia [33]. Adoptive T cell therapy represents an advancement for personalized medicine in the form of customized cellular therapies. However, multiple challenges will have to be addressed prior to these technologies becoming commercially available and offered as a standard of care. Efforts are currently underway to demonstrate that adoptive T cell therapy is clinically efficacious, safe, reproducible, perhaps most importantly, exportable beyond a limited range of academic centers.

### Anti-tumor antibodies

Monoclonal antibodies (mAb) directed against tumor associated antigens like CD20 and HER-2 are a standard of care treatment in many malignancies. This technology was facilitated by the simultaneous understanding of antibody structure and the application of hybridoma technology, leading to a Nobel Prize for Jerne, Kohler, and Milstein in 1984. Antibodies are highly specific agents, and knowledge of their structure and potential modifications plays an increasingly important role in cancer immunotherapy. There is an extremely diverse but highly specific region (with potentially low nanomolar affinity) called the Fab region which binds antigen fragments. In addition, the Fc region (constant domain) controls the host immune response. Though there are a variety of antibody subtypes, in the context of IgG antibodies (used primarily for therapeutics in oncology) there are four Fc gamma receptors (FcγR) in humans. In general the Fc portion of an antibody can interact with Fc receptors on cells such as natural killer (NK) cells, thus promoting mAb bound target cell lysis through a process known as antibody-dependent cellular cytotoxicity (ADCC). Monoclonal antibodies can also mediate complement-dependent cytotoxicity (CDC) by directly activating the complement cascade and membrane attack complex (MAC). CDC generally requires antibody crosslinking, and this approach is rarely used in the development of mAbs for cancer therapy. IgG1 mAb subtypes can often have the most significant ADCC, whereas mAbs of the human IgG4 subtype are thought to function primarily as agonists (signaling) or antagonists (blocking) with minimal ADCC, especially if Fc region glycoslyation is eliminated.

Over the past few years a number of modified antibody technologies have emerged, including radioimmunotherapies, TRAP molecules, antibody-drug conjugates (ADCs), single chain dual specificity bi-specific T cell engagers (BiTEs), and chimeric antigen receptors (CARs). Indeed, as early as 2002 the FDA approved radioimmunoisotopes to treat refractory non-Hodgkin’s lymphoma with agents such as ibritumomab (anti-CD20 + Yttrium-90) and in 2003 tositumomab (anti-CD20 + Iodine 131). In 2012 the FDA approved the use of aflibercept (a TRAP molecule combining 2 separate regions mimicking VEGFR1 and VEGFR2 bound to an IgG1 Fc region) for metastatic colorectal carcinoma. There has also been a great deal of excitement about the development of antibody-drug conjugates, as these agents are designed to improve local delivery of highly toxic chemotherapeutics while simultaneously attempting to minimize systemic toxicity. In 2011 the FDA approved brentuximab vedotin (anti-CD30-MMAE [monomethyl auristatn E]) for relapsed or refractory Hodgkin lymphoma or anaplastic large cell lymphoma and in 2013 approved trastuzumab emtansine (TDM-1) for patients with metastatic HER2 positive breast cancer.

Another interesting technology has been the development of engineered bi-specific antibodies, in which one fragment arm binds the CD3 portion of the T cell receptor (TCR) on T lymphocytes, and the other fragment arm carries specificity for a tumor antigen. These constructs aim to co-localize T lymphocytes to tumor cells and thus induce anti-tumor immune responses. Blinatumomab (a BiTE specific for the B cell surface marker CD19) is currently undergoing phase I and II clinical trials in non-Hodgkin’s lymphoma and acute lymphoblastic leukemia (ALL) among other diseases [34,35]. Though discussed in detail within the review of adoptive T cell therapy, the engineering of T cells to express chimeric antigen receptors (CARs) for cancer immunotherapy is also an antibody based targeting modality. In addition to a variety of targets that CARs have been designed (including CD19), one of the interesting newer applications for this technology is based on detailed preclinical work for targeting the EGFRvIII splice variant in human Glioblastoma Multiforme (GBM). Based on rapid development of this technology, clinical trials in patients with recurrent GBM utilizing EGFRvIII CARs have already been initiated at the NIH, and trials for newly diagnosed patients (after initial standard of care surgery and concurrent radiation with chemotherapy) may open in 2014 [36,37]. Indeed, it is likely that monoclonal-antibody based immunotherapies will likely represent an increasing portion of the oncology portfolio in the years to come.

### Obstacles to driving an immune response

The immune system is extremely potent, as evidenced by auto-immune disease or cytokine storm. In order to rapidly expand and respond to pathogens there are inherent positive feedback loops which facilitate activation. However, in order to maintain homeostasis and prevent the potentially dangerous complications of excessive immune activation, there are arguably even more powerful negative regulatory feedback loops. Indeed, there are a variety of different mechanisms used to suppress the immune response including physical barriers, loss of antigen or MHC, increasing expression of inhibitory cell surface molecules, secretion of inhibitory cytokines, and recruitment of suppressor cell populations. Unfortunately, aggressive tumor cells hijack these mechanisms to evade the immune system and this presents obstacles to the initiation and propagation of a successful anti-tumor immune response.

The concept of immune surveillance was initially proposed by Burnet and Thomas. Immune surveillance proposes that the immune system is constantly screening and potentially eliminating aberrant or transformed cancer cells. Robert Schreiber and colleagues brought the concept back to the forefront in 2004 with the three E's of cancer immunoediting: Elimination, Equilibrium, and Escape [1]. Elimination is the initial phase of immune surveillance where the innate and adaptive immune systems work together to destroy and control malignant cells. However, there is a proposed state of equilibrium at a point where cancer cells have accumulated sufficient alterations to resist immune mediated cell death and survive at a steady state. At some point subsequent to this the cancer cells continue to suppress the immune system to the point of it becoming refractory and reach the escape phase where they rapidly proliferate and spread.

One of the most fundamental obstacles to immune function is a physical barrier or immune-privileged site, which is defined as a physiological location in which cells of the immune system have limited access and where the immune system is restricted or suppressed by physical as well as molecular mechanisms. There are four main immune-privileged sites in the human body: the brain, the eye, the intrauterine fetus/placenta, and the testes. The purpose of these immune-privileged sites is to protect delicate and sensitive structures, which may also harbor potentially foreign or immunogenic antigenic epitopes from destruction by the immune system. In the case of the blood–brain barrier (BBB) this is certainly not absolute as inflammation can increase the permeability of the BBB, yet it remains an important consideration when using a systemic immunotherapy to treat intracranial disease.

Metastatic melanoma provides ample evidence for the processes involved in immune-editing [38]. For example, malignant melanoma is known to have low basal levels of HLA. Additionally, metastatic melanoma patients have been observed to have increased quantities of myeloid derived suppressor cells (MDSC) [39]. These CD14+ MDSC were also shown to directly suppress T-cell proliferation ex-vivo [39]. One of the inherent obstacles when attempting to elicit an immune response against tumor antigens is that many of the antigens will be self antigens to which high avidity T-cells have been previously deleted or anergized via central or peripheral tolerance. Nevertheless, effective T-cell mediated immune responses with clinical benefit can be mounted against tumor associated melanoma antigens including tyrosinase, MART-1/Melan-A, and mutated CDK4. One interesting strategy to overcome tolerance to self antigens is xenogeneic DNA immunization, whereby xenogeneic DNA from a mouse, for example, is used to prime immunity by diversifying the antigenic epitopes [40,41].

One of the now well known regulatory mechanisms which serve to dampen or shut down T-cell responses are immune checkpoint molecules expressed on the T-cell surface, including CTLA-4 and PD-1. An area of active investigation is the dynamic ability of tumor cells to up-regulate or express ligands for these checkpoints such as PD-L1 or PD-L2. Clearly a combinatorial approach will be needed to overcome the multiple layers of negative regulation, which could include combined checkpoint blockade [42], adoptive T-cell transfer with lymphablation, or incorporating other modalities such as radiotherapy, which has been shown to upregulate MHC expression and increase susceptibility to immune mediated cell death [43].

### Cancer vaccines

Cancer vaccines, like the conventional vaccines used to prevent infectious diseases, generally involve inoculating a patient with a reagent designed to induce an antigen specific immune response. Infectious disease vaccines, though, are preventative vaccines which rely upon priming the adaptive immune response to generate a memory response which can more rapidly expand upon pathogen exposure and prevent full-blown infection. In the setting of cancer, oncogenic viruses are an ideal target for preventative cancer vaccines, and the HPV vaccine has been shown in large clinical trials to drastically reduce the chances of developing cervical cancer [44]. Importantly, multiple other tissues such as oral and anal mucosa are susceptible to HPV mediated transformation, and thus the HPV vaccine has the potential to reduce development of multiple different types of cancer. Other examples of preventative cancer vaccines include the HBV vaccine which can significantly reduce the incidence of hepatocellular carcinoma [45]. Therapeutic cancer vaccines, on the other hand ,aim to treat cancer after diagnosis. This task can be much more difficult given the development of immune tolerance mechanisms and the obstacles to immune function as described above. In general, several broad categories of therapeutic cancer vaccines include peptide based vaccine, cell-based vaccines, virus-based vaccine and vaccines based on ex-vivo generated dendritic cells. This is a broad topic, which has been well covered in several recent reviews [46,47].

### Immune checkpoint blockade

Probably due primarily to recent clinical success [48-50] a great deal of excitement in immunotherapy has surrounded further understanding and modulating immune checkpoints for cancer immunotherapy. As described earlier, when a T cell interacts with an antigen presenting cell (APC) through the TCR-antigen/MHC complex, there are both costimulatory and coinhibitory signals occurring simultaneously that ultimately affect downstream T cell responses. Costimulation classically involves the interaction of B7 with CD28, and disruption of this interaction by the presence of CTLA-4 on the surface of T cells is one example of coinhibition [51,52]. Early work in the field led by Allison and colleagues showed that in preclinical models blockade of CTLA-4 induces an anti-tumor immune response [53]. This initial body of work culminated in a phase III trial in which CTLA-4 blockade with an anti-CTLA-4 mAb improved overall survival in patients with metastatic melanoma compared to patients receiving a tumor vaccine [54], and to subsequent approval of the anti-CTLA-4 antibody ipilimumab for metastatic melanoma [55].

Tumor infiltrating lymphocytes can express, in addition to CTLA-4, other immune checkpoint molecules such as PD-1, LAG-3, and TIM-3 among others [56-58]. As discussed above, PD-1 inhibition appears to have clinical activity in a variety of cancers, showing durable responses in a proportion of patients, many of whom failed other therapies [48,49]. In addition, combining CTLA-4 blockade with PD-1 blockade (ipilimumab + nivolumab) in metastatic or advanced melanoma patients showed a large proportion of patients with dramatic and rapid responses in their disease burden with a proportion of patients maintaining a durable response [59]. These checkpoint inhibitors are also being tested in a variety of tumor types including non-small cell lung carcinoma (NSCLC), small cell lung carcinoma (SCLC), renal cell carcinoma (RCC), prostate cancer, and hematological malignancies. Further emphasizing interest in combined checkpoint blockade, a phase I trial combining PD-1 and LAG-3 inhibition has recently opened. Other combination approaches include combining immune checkpoint blockade with chemotherapy (cytotoxic or low dose regimens), or other immune-modulating therapies (including cytokines such as IL-2 or IL-21, cell based vaccines, peptide vaccines, or indolamine 2–3 dioxygenase inhibitors), molecularly targeted therapies (JAK/STAT inhibitors, BRAF inhibitors), and radiation therapy (stereotactic radiation versus conventional or intensity modulated radiation therapy). In particular, combining radiation therapy with immunotherapy is an area of intense clinical and pre-clinical research activity, incited to some degree by a recent case report of a potential abscopal effect in a patient with metastatic melanoma [60]. Continued delineation of the most effective combinatorial approaches for patients is important, as optimal combinations will likely be different for various tumor types.

### Monitoring T cell and B cell responses

Immune monitoring can define immune correlates of clinical responses and delineate the specificity of anti-tumor immune responses induced by various forms of immunotherapy. More broadly, monitoring can encompass multiple fields including immunology, pathology, genomics, proteomics, and imaging. Techniques for monitoring T cells and B cells include quantification of cytokine release in the supernatant by ELISA, CTL chromium release assay, CD4 T-helper cell thymidine incorporation proliferation assay, and traditional ELISA assays to detect antibody titers. At a more cellular level, techniques include ELISPOT assays, flow cytometric analyses, tetramer staining, and intracellular staining for cytokines. Multiplexed assays, sequencing, and array based technologies can also be used to screen more broadly for potential immune responses. These higher throughput methods include phenotyping of populations, multiplex cytokine arrays, immunogenomics of T-cells and B-cells, sequencing of TCR and BCR, seromics assays to profile antibodies, and immunohistochemistry and imaging of T-cells and B-cells.

When selecting assays to monitor immune responses one must address the question of whether the immune response is readily detectable. For example, it may be difficult to detect NY-ESO-1 CD8+ T-cell responses directly from ex-vivo peripheral blood and in-vitro re-stimulation methods can be used to increase the detectable percentage and activation of antigen specific cells [61]. Another question is whether to draw samples for immune monitoring from the peripheral blood versus locally in-situ. Given the specificity and compartmentalization of the immune system the source of sample for immune monitoring can be critical for proper readout and interpretation of the assay.

Correlating specific immune responses with clinical outcomes is an aspirational goal for immune monitoring. Such successful correlative studies are relatively rare, but include an association between IFN-gamma response to vaccine and survival in a trial of an autologous DC lysate vaccine in GBM [62]. In a series of therapeutic vaccine trials for renal cell cancer, patients who received single dose cyclophosphamide and manifested a multipeptide immune response to the IMA901 vaccine had a longer survival than those with no detectable immune responses or single peptide immune responses [63]. These studies highlight the potential relevance of immune monitoring when using immunotherapy, as the clinical data gained from analyzing patient responses could be critical to guiding scheduling, dosing, and optimal incorporation of immunotherapy into current treatment paradigms.

### Clinical trial design for immune monitoring

Cancer immunotherapies, despite being attractive and potentially curative treatment strategies, have not demonstrated sufficient clinical activity to justify routine use in most malignancies. Many challenges exist in the field including the difficulty assessing clinical response due to delayed response kinetics, the lack of biomarkers, questions regarding optimal dosing and scheduling of various therapies, and potentially inappropriate patient selection. Patent selection may be of particular importance in early Phase I studies, which typically enroll heavily pre-treated patients who might be less likely to benefit from immunotherapy. One approach to overcoming these challenges is to perform phase IA and IIA pre-surgical trials – which are often called neo-adjuvant studies. The primary goal of a pre-surgical clinical trial is to collect tissue and blood for biomarker analysis in order to provide mechanistic insights into the immunotherapy utilized in that trial. These trials are hypothesis-generating endeavors that use a discovery-driven approach in order to bring clinical questions to the lab and then back again to the clinic [64]. For example, at the MD Anderson Cancer Center, pre-surgical trials in bladder and prostate cancer have provided insights into the mechanism of action of anti-CTLA-4 therapy. In patients with localized bladder cancer who were treated with preoperative ipilimumab, CD4^+^ T cells from the peripheral blood and tumor tissues were found to have increased expression of ICOS (inducible costimulator) compared to patients who did not receive ipilimumab. These CD4^+^ICOS^hi^ T cells produced IFN-gamma and could recognize the tumor antigen NY-ESO-1 [65]. This was the first study reporting immunological changes in both tumor tissues and peripheral blood after treatment with anti-CTLA-4 therapy. Furthermore, in a small retrospective study, an increased frequency of CD4^+^ICOS^hi^ T cells sustained over a period of 12 weeks of therapy correlated with increased likelihood of overall survival benefit in a cohort of patients with melanoma [66]. CD4^+^ICOS^hi^ T cells were also shown to have a specificity of ~96% as a pharmacodynamic biomarker for anti-CTLA-4 therapy [67]. In animal studies, ICOS was found to be necessary for optimal anti-tumor responses with anti-CTLA-4 [68] and ICOS was shown to mediate PI3K-signaling to increase T-bet expression in the setting of anti-CTLA-4 therapy [69]. Indeed, targeting ICOS may lead to improved anti-tumor responses with anti-CTLA-4 therapy. Thus, a preoperative clinical trial model is a powerful tool that can help identify biomarkers and other molecular pathways that can be targeted in combination with currently approved agents.

## Conclusions

Detailed knowledge of the function of the immune system has been increasing dramatically over the past decade. This has led to the understanding that not only is the immune system able to identify non-self from self, but that it also may recognize “altered-self” in the setting of cancer development. Though these relationships may play a role in suppressing the formation or progression of certain tumors, there are clearly scenarios in which endogenous anti-tumor immune responses are inhibited through a variety of mechanisms.

Thus, a continued exploration of the workings of the innate and adaptive immune systems is paramount to utilizing therapeutic techniques for cancer immunotherapy. There have been a number of recent advancements in therapeutic approaches utilizing dendritic cells, cancer vaccines, anti-tumor antibodies, adoptive T cell therapy, immune checkpoint blockade, and combinations of these strategies with other modalities such as chemotherapy or radiation therapy. In addition, there are still many challenges and obstacles to overcome in fully realizing the potential of immunotherapy, and there are also important implications for the future of clinical trial design as well in the era of personalized medicine.

Some of the approaches reviewed here have led to groundbreaking medical advancements for many cancer patients. This shared goal of ultimately improving outcomes for patients is the impetus behind educating researchers and clinicians through the SITC Primer. Indeed, a more detailed understanding of the mechanisms of the immune system and its interaction with the tumor microenvironment is central to developing effective therapeutic strategies for cancer immunotherapy.

## Competing interests

PS has ownership interest (including patents) in Jounce and served as a paid consultant/advisory board member for Bristol Myers Squibb (BMS) and MedImmune. CGD has served as a paid consultant for Amplimmune, Bristol Myers Squibb (BMS), Compugen, CoStim, Dendreon, ImmunExcite, and Roche/Genentech. No potential competing interests were disclosed by the other authors.

## Authors’ contributions

RR, AS, and AW performed the literature search; drafted and edited the manuscript; and RR incorporated all edits from all authors into the manuscript. PS and CD supervised the preparation of the manuscript and contributed background research and writing for the manuscript. All authors read and approved the final manuscript.

## Authors’ information

Raju R. Raval, MD DPhil, is currently a senior resident in Radiation Oncology at the Johns Hopkins University School of Medicine and will be joining the faculty at The Ohio State University as an Assistant Professor of Radiation Oncology in 2014. He has received prior research support from an ASCO Young Investigator Award, the Johns Hopkins Brain Science Institute, the Rhodes Scholarship, and through an NIH CRTA. Andrew B. Sharabi, MD PhD, is a resident in Radiation Oncology at the Johns Hopkins University School of Medicine. He has conducted research within the laboratory of Dr. Charles Drake, and has received research support from an ASTRO Resident Research Seed Grant and the MSTP while at the Baylor College of Medicine. Amanda J. Walker, MD, is a resident in Radiation Oncology at the Johns Hopkins University School of Medicine and has received prior research support from an RSNA Research Resident Grant and through an NIH CRTP. Charles G. Drake, MD PhD, is an Associate Professor of Medical Oncology, Immunology, and Urology at the Johns Hopkins University School of Medicine and serves as the co-director of the multi-disciplinary prostate cancer clinic. He has focused research efforts in his laboratory and in clinical trials on cancer immunotherapy. He is a Damon Runyon-Lilly Clinical Investigator and is supported by National Institutes of Health R01 CA127153, 1P50CA58236-15, the Patrick C. Walsh Fund, the One-in-Six Foundation, the Koch Foundation and the Prostate Cancer Foundation. Padmanee Sharma, MD PhD, is an Associate Professor of Genitourinary Medical Oncology and Immunology at The University of Texas MD Anderson Cancer Center and serves as the Scientific Director of the Immunotherapy Platform at the institution. Her preclinical research and interest in clinical trials also focuses on cancer immunotherapy, and she has received research funding and support from a variety of sources including an NIH R01 Award, a CPRIT Award, a DOD/CDMRP Idea Development Award, an ACS Research Scholar Grant, a Doris Duke Clinical Scientist Development Award, and a Prostate Cancer Foundation Challenge Award in Immunology.
